# Prognostic role of decreased E-cadherin expression in patients with upper tract urothelial carcinoma: a multi-institutional study

**DOI:** 10.1007/s00345-016-1835-1

**Published:** 2016-04-29

**Authors:** Ricardo L. Favaretto, Atessa Bahadori, Romain Mathieu, Andrea Haitel, Bernhard Grubmüller, Vitaly Margulis, Jose A. Karam, Morgan Rouprêt, Christian Seitz, Pierre I. Karakiewicz, Isabela W. Cunha, Stenio C. Zequi, Christopher G. Wood, Alon Z. Weizer, Jay D. Raman, Mesut Remzi, Nathalie Rioux-Leclercq, Solene Jacquet-Kammerer, Karim Bensalah, Yair Lotan, Alexander Bachmann, Michael Rink, Alberto Briganti, Shahrokh F. Shariat

**Affiliations:** 1 Department of Urology, A. C. Camargo Hospital, São Paulo, Brazil; 2 Department of Urology and Comprehensive Cancer Center, Medical University of Vienna, Vienna General Hospital, Währinger Gürtel 18-20, 1090 Vienna, Austria; 3 Department of Urology, Rennes University Hospital, Rennes, France; 4 Department of Pathology, Medical University Vienna, Vienna, Austria; 5 Department of Urology, University of Texas Southwestern Medical Center at Dallas, Dallas, TX USA; 6 Department of Urology, MD Anderson Cancer Center, Houston, TX USA; 7 Academic Department of Urology, Faculté de Médecine Pierre et Marie Curie, La Pitié-Salpetrière Hospital, Assistance Publique-Hôpitaux de Paris, University Paris 6, Paris, France; 8 Cancer Prognostics and Health Outcomes Unit, University of Montreal Health Centre, Montreal, Canada; 9 Department of Urology, University of Michigan Cancer Center, Ann Arbor, MI USA; 10 Division of Urology, Penn State Milton S. Hershey Medical Center, Hershey, PA USA; 11 Department of Pathology, Rennes University Hospital, Rennes, France; 12 Department of Urology, University Hospital Basel, Basel, Switzerland; 13 Department of Urology, University Medical Center Hamburg-Eppendorf, Hamburg, Germany; 14 Department of Urology, Vita Salute San Raffaele University, Milan, Italy; 15 Department of Urology, Weill Cornell Medical College, New York, NY USA

**Keywords:** E-cadherin, Urothelium, Carcinoma, Recurrence, Prognosis, Survival, Prediction

## Abstract

**Purpose:**

To assess the role of E-cadherin as prognostic biomarker in upper tract urothelial carcinoma (UTUC) in a large multi-institutional cohort of patients.

**Methods:**

Immunohistochemistry technique was used to evaluate E-cadherin expression in 678 patients with unilateral, sporadic UTUC treated with RNU. E-cadherin expression was considered decreased if 10 % or more cells had decreased expression (<90 %).

**Results:**

Decreased E-cadherin expression was observed in 353 patients (52.1 %) and was associated with advanced pathological stage (*P* < 0.001), higher grade (*P* < 0.001), lymph node metastasis (*P* = 0.006), lymphovascular invasion (*P* < 0.001), concomitant carcinoma in situ (*P* < 0.001), multifocality (*P* = 0.004), tumor necrosis (*P* = 0.020) and sessile architecture (*P* < 0.001). Within a median follow-up of 30 months (interquartile range 15–57), 171 patients (25.4 %) experienced disease recurrence and 150 (21.9 %) died from UTUC. In univariable analyses, decreased E-cadherin expression was significantly associated with worse recurrence-free survival (*P* < 0.001) and cancer-specific survival CSS (*P* = 0.006); however, in multivariable analyses, it was not (*P* = 0.74 and 0.84, respectively). The lack of independent prognostic value of E-cadherin remained true in all subgroup analyses.

**Conclusion:**

In UTUC patients treated with RNU, decreased E-cadherin expression is associated with features of biologically and clinically aggressive disease and worse outcome in univariable, but not multivariable, analyses. If E-cadherin’s association with factors of advanced disease is confirmed on UTUC biopsy specimens, it could be used to help in the clinical decision-making regarding kidney-sparing approaches and/or neo-adjuvant chemotherapy.

## Introduction

 Upper tract urothelial carcinoma (UTUC) is a rare disease, accounting for 5–10 % of all urothelial carcinomas [1]. In the last two decades, management of UTUC has improved but still remains challenging. Radical nephroureterectomy (RNU) remains the standard treatment for non-metastatic disease [1, 2]. However, kidney-sparing approaches are now considered for low-risk UTUC, and conversely, regional lymphadenectomy and perioperative chemotherapy are discussed in addition to RNU for high-risk UTUC [1, 2]. Current concerns lay in the identification of the patients who may benefit from these treatments. Recent evidence suggests that carcinogenetic mechanisms in UTUC are different from urothelial carcinoma of the bladder (UCB) [2–4]. Therefore, molecular alterations from one setting may not be extrapolated in the other. In this regard, specific validation of biomarkers in UTUC is mandatory to develop predictive tools that could allow accurate clinical decision-making in the management of UTUC patients.

Decreased expression of the membrane-associated glycoprotein E-cadherin has been established as a feature of epithelial–mesenchymal transition (EMT) in epithelial malignancies [5–7]. In normal cells, E-cadherins’ cytoplasmic domain binds with subtypes β or γ of the catenin proteins, which in turn secure attachment to the actin microfilament, thus ensuring cytoskeleton integrity and stable cellular adhesion [8]. Loss of cellular adhesion is a tipping point in tumor progression resulting in poorly differentiated and invasive tumors [7, 8]. E-cadherin has been shown to be an independent prognostic factor in UCB [3, 9]. In UTUC, single-center studies with small cohorts have investigated the role of E-cadherin expression with conflicting results [10–15]. We hypothesized that E-cadherin expression in RNU specimens was associated with features of biologically and clinically aggressive UTUC, thereby potentially helping in the clinical decision-making of UTUC patients. To assess this hypothesis, we tested the association of E-cadherin with pathologic characteristics and prognosis in a large multi-institutional cohort of patients treated by RNU for UTUC.

## Materials and methods

### Patient selection

This was a retrospective, institutional review board-approved study involving seven institutions from the international UTUC collaboration [16]. The initial study cohort comprised 753 patients who underwent RNU for UTUC (Ta–T4 N0–1 M0) between March 1990 and May 2008. Exclusion criteria included neo-adjuvant chemotherapy/radiotherapy and follow-up <3 months, resulting in a final cohort of 678 patients.

### Data collection, pathological evaluation and immunochemistry

A computerized database was used to collect patient and tumor characteristics. All surgical specimens were processed according to standard pathological procedures. Original pathology slides were centrally collected and analyzed by genitourinary pathologists blinded to clinical outcome. Pathological stage was determined according to the 2002 tumor, node and metastasis (TNM) staging system, and the pathological grading using the 1998 WHO/ISUP consensus classification. The tumors were architecturally defined as papillary or sessile [17]. The presence of tumor cells within an endothelium-lined space without underlying muscular walls was defined as lymphovascular invasion (LVI) [18]. Multifocal tumor [19], carcinoma in situ and tumor necrosis [20] were confirmed in every slide.

**Fig. 1:**
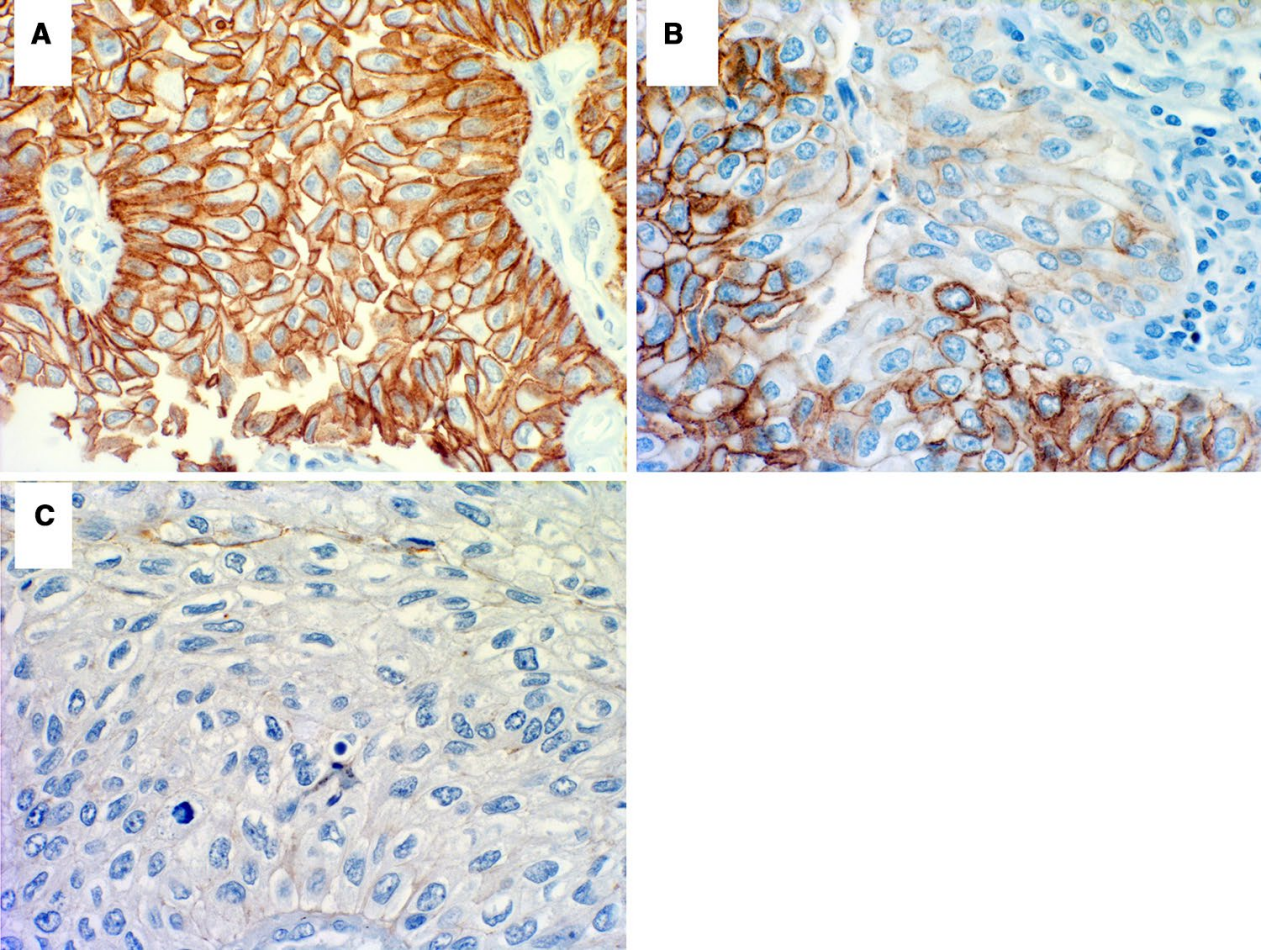
E-cadherin immunohistochemical staining of upper tract urothelial carcinoma: **a** normal expression (range 90–100 %), **b** heterogenous expression (range 11–89 %) and **c** absent expression (range 0–10 %)

E-cadherin staining was performed on formalin-fixed tissue microarray slides constructed for the study in a single laboratory, as described previously [9]. Antigen retrieval was performed and the primary anti-E-CD monoclonal mouse antibody (Transduction Labs, dilution 1:25 in blocking solution) was incubated for 1 h. Secondary antibody (Vector Labs) was applied at a dilution of 1:400. Reactivity was visualized with an avidin–biotin complex immunoperoxidase system using diamino benzidine as the chromogen and methyl green and alcian blue as the counterstain. Positive controls included bladder and prostate tissue known to possess 100 % preserved E-cadherin expression (external control) and normal urothelium (internal control) included in cancer specimens. Negative controls were serial sections processed without incubation in primary antibody. Areas of urothelial tumor were classified as normal (90–100 % cells with preserved cell border staining resembling membranous staining of normal controls) and abnormal, which included negative (0–10 % positive tumor cells) and various degrees of heterogeneous decreased expression (11–89 % positive tumor cells) (Fig. 1). Multiple sections from the same patient were evaluated to minimize the effect of the staining technique on interpretation. The negative and the heterogeneously staining tumors were considered together in statistical calculations based on the premise that the negative areas of the heterogeneous tumors would define the biological behavior of the tumor as a whole [9, 21].

### Management and follow-up

All patients underwent standard RNU [1]. Additionally, a regional lymphadenectomy was performed in 155 patients (22.9 %) and 68 patients (10 %) received adjuvant chemotherapy. Postoperative follow-up was generally performed every 3 months the first year after surgery, every 6 months in the second year and annually thereafter. Relapse was defined by local recurrence or distant metastasis. Cause of death was determined by chart review or death certificate [22].

### Statistical analyses

Outcomes included recurrence-free survival (RFS) and cancer-specific survival (CSS). Chi-square test was used to assess decreased E-cadherin expression with categorical variables. Differences in continuous variables were analyzed using Kruskal–Wallis tests. The Kaplan–Meier method was used to estimate RFS and CSS; log-rank tests were applied for pairwise comparison of survival. Univariable and multivariable Cox regression models addressed associations of RFS and CSS with potential prognostic factors. We performed subgroup analyses in patients with pTa–pT4 high-grade disease, pTa–pT2 N0/Nx, pT1–pT3 N0/Nx, pT3/pT4 N0/Nx, pTa–pT4 pN0 and pTa–pT4 pN1 disease. All P values were two-sided, and statistical significance was defined as *P* < 0.05. Statistical analyses were performed using Stata 11.0 statistical software (StataCorp., College Station, TX, USA).

## Results

### Descriptive characteristics and association with pathology

Decreased E-cadherin expression was observed in 353 patients (52.1 %). There was a significant association between decreased E-cadherin expression and pathological adverse features such as advanced pathological tumor stage (*P* < 0.001), high pathological tumor grade (*P* < 0.001), lymph node metastases (*P* = 0.006), LVI (*P* < 0.001), concomitant carcinoma in situ (*P* < 0.001), multifocality (*P* = 0.004), tumor necrosis (*P* = 0.020) and sessile architecture (*P* < 0.001) (Table 1).

**Table 1:** Association of decreased E-cadherin expression with clinicopathological characteristics in 678 patients treated with radical nephroureterectomy for upper tract urothelial carcinoma

	All patients	Normal E-cadherin	Decreased E-cadherin	*P*
Total, *n* (%)	678	325 (47.9)	353 (52.1)	
Age (years)				0.19
Median (IQR)	69 (63–76)	69 (62–76)	70 (63–77)	
Gender, *n* (%)				0.78
Male	380 (56.1)	184 (56.6)	196 (55.5)	
Female	298 (43.9)	141 (43.4)	157 (44.5)	
Tumor stage, *n* (%)				<0.001
pTa	121 (17.8)	82 (25.2)	39 (11.1)	
pT1	208 (30.7)	98 (30.1)	110 (31.2)	
pT2	123 (18.1)	60 (18.5)	63 (17.8)	
pT3	193 (28.5)	74 (22.8)	119 (33.7)	
pT4	33 (4.9)	11 (3.4)	22 (6.2)	
Grade, *n* (%)				<0.001
Low	174 (25.6)	114 (35.1)	60 (17.0)	
High	504 (74.3)	211 (64.9)	293 (83.0)	
Lymph node status, *n* (%)				0.006
pNx	523 (77.2)	261 (80.3)	262 (74.2)	
pN0	108 (15.9)	52 (16)	56 (15.9)	
pN1	47 (6.9)	12 (3.7)	35 (9.9)	
Lymphovascular invasion, *n* (%)				<0.001
Yes	135 (19.9)	26 (8.0)	109 (30.9)	
No	543 (80.1)	299 (92.0)	244 (69.1)	
Concomitant carcinoma in situ, *n* (%)				<0.001
Yes	128 (18.9)	41 (12.6)	87 (24.7)	
No	550 (81.1)	284 (87.4)	266 (75.3)	
Multifocality, *n* (%)				0.004
Yes	145 (21.4)	54 (16.6)	91 (25.8)	
No	533 (78.6)	271 (83.4)	262 (74.2)	
Necrosis, *n* (%)				0.020
Yes	81 (11.9)	29 (8.9)	52 (14.7)	
No	597 (88.1)	296 (91.1)	301 (85.3)	
Architecture, *n* (%)				<0.001
Papillary	558 (82.3)	293 (90.1)	265 (75.1)	
Sessile	120 (17.7)	32 (9.9)	88 (24.9)	
Location				0.70
Kidney	478 (70.5)	232 (71.4)	246 (69.7)	
Ureter	200 (29.5)	93 (28.6)	107 (30.3)	

### Association of decreased E-cadherin expression with cancer recurrence and cancer-specific survival

**Fig. 2:**
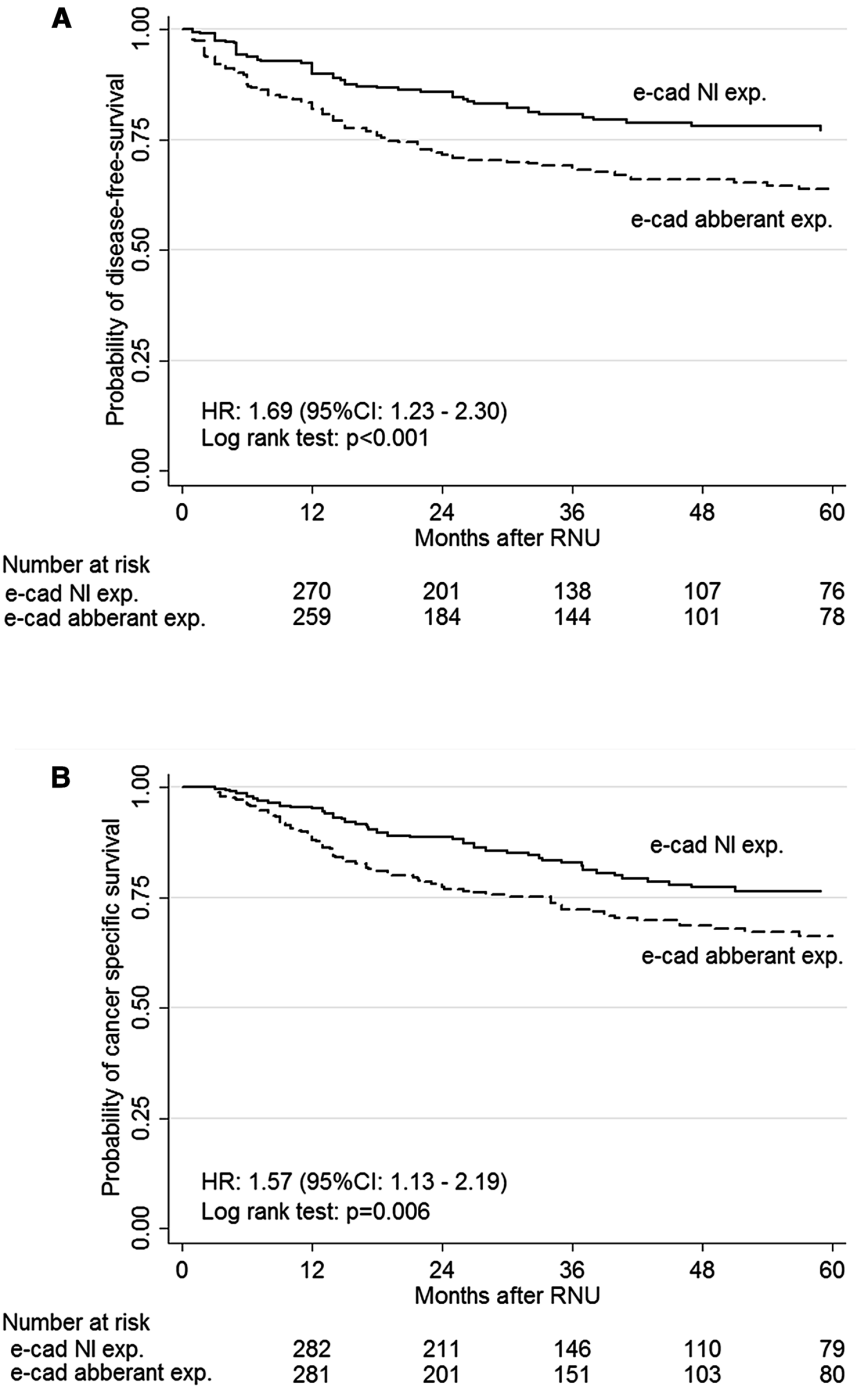
Kaplan–Meier estimates of disease-free survival (**a**) and cancer-specific survival (**b**) according to the expression of E-cadherin in 678 patients treated with radical nephroureterectomy for upper tract urothelial carcinoma

The median follow-up time was 30 months (15–57). Within this period, 171 patients (25.4 %) experienced disease recurrence and 150 (21.9 %) died from UTUC. In univariable analyses, decreased E-cadherin expression was associated with a higher probability of disease recurrence (log-rank test *P* < 0.001, HR 1.69, 95 % CI 1.23–2.30) (Fig. 2a) and cancer-specific mortality (log-rank test *P* = 0.006, HR 1.57, 95 % CI 1.13–2.19) (Fig. 2b). Table 2 summarizes the Cox regression analyses. In multivariable analyses, decreased E-cadherin expression was not associated independently with either RFS (HR 1.06, *P* = 0.74) or CSS (HR 0.96, *P* = 0.84) (Table 2).

**Table 2:** Multivariable Cox regression analyses predicting disease recurrence and cancer-specific mortality of 678 patients treated with radical nephroureterectomy for upper tract urothelial carcinoma

	Disease-free survival			Cancer-specific survival		
	HR	95 % CI	*P* value	HR	95 % CI	*P* value
Male gender	0.76	0.56–1.04	0.085	0.78	0.56–1.08	0.13
Age	1.02	0.99–1.03	0.052	1.03	1.01–1.04	0.006
Pathological stage	Ref.			Ref.		
Ta						
T1	1.80	0.76–4.28	0.184	1.48	0.57–3.80	0.42
T2	3.58	1.52–8.44	0.003	3.49	1.40–8.73	0.008
T3	7.31	3.21–16.65	<0.001	6.29	2.58–15.31	<0.001
T4	32.98	12.81–84.87	<0.001	25.16	9.11–69.53	<0.001
Pathological high grade	1.38	0.82–2.32	0.23	1.59	0.89–2.82	0.12
Lymphovascular invasion	1.14	0.80–1.64	0.47	1.31	0.89–1.92	0.16
Concomitant CIS	1.52	1.03–2.23	0.035	1.06	0.69–1.63	0.78
Architecture	1.27	0.86–1.86	0.23	1.35	0.89–2.06	0.15
Necrosis	0.46	0.29–0.75	0.002	0.51	0.30–0.85	0.009
Multifocality	1.44	1.01–2.056	0.045	1.78	1.22–2.58	0.003
Kidney location	1.17	0.86–1.60	0.32	1.26	0.90–1.76	0.18
Lymph node metastasis	2.40	1.57–3.66	<0.001	2.28	1.46–3.55	<0.001
E-cadherin decreased expression	1.06	0.76–1.48	0.74	0.96	0.68–1.38	0.84

*CI* confidence interval, *CIS* carcinoma in situ, *HR* hazard ratio

Further univariable analyses in subgroups of patients revealed that decreased E-cadherin expression was associated with worse outcomes in patients with pTa–pT4 M0 high-grade tumors (HR 1.55, *P* < 0.011) and pTa–pT2 pN0/Nx M0 tumors (HR 2.20, *P* < 0.038) regarding RFS and in patients with pTa–pT4 M0 high-grade tumors (HR 1.50, *P* < 0.025) regarding CSS. However, in these subgroups, the prognostic value of E-cadherin did not retain statistical significance when adjusted for the effects of standard clinicopathological features.

## Discussion

In this study, we assessed the clinical significance of a decreased E-cadherin expression in an international cohort of 678 UTUC patients treated with RNU. We found that decreased E-cadherin expression in tumor cells is associated with adverse clinicopathological features and worse outcomes.

Half of the patients in this cohort presented with decreased expression of E-cadherin in the tumor. This was within the range previously reported in UCB patients (31–77 %) [8, 9] but lower than that reported in UTUC patients (68–71 %) [10, 11]. This could be due to our lower proportion of high stage tumors compared to the two other studies (50 vs. 54 and 61 %), as well as methodological differences in scoring, staining protocols, choice of antibody and/or antigen retrieval.

Patients with decreased E-cadherin were most likely to harbor tumors with features of biologically aggressive disease. This association is in line with the biological role of E-cadherin, as a calcium-dependent glycoprotein essential to epithelial tissue integrity. Loss of cellular adhesion results in the detachment of cancerous cells from the primary lesion, promoting invasiveness [23]. In carcinoma in situ of the bladder, for example, loss of E-cadherin expression predicts RFS, disease progression and CSS [21]. Similar results were reported in various UCB studies [8, 9, 24–27] and one UTUC study [10]: loss of E-cadherin immunoreactivity strongly correlated with advanced stage and high-grade tumors.

We further evaluated the relevance of E-cadherin as a biomarker to predict outcomes after RNU. The role of E-cadherin expression as a prognostic factor in urothelial carcinoma was supported by previous studies mainly focusing on UCB [8, 9, 24–27]. Our results confirm that decreased E-cadherin expression is indeed associated with a higher probability of disease recurrence and cancer-specific mortality in UTUC. However, when adjusted for the effects of established prognostic factors in multivariable analyses, E-cadherin expression lost its independent prognostic value and, therefore, may have only limited value in clinical practice. Previous studies that addressed the relationship between E-cadherin and outcomes in UTUC led to conflicting results. Fromont et al. [14] showed, in a cohort of 62 UTUC patients, that decreased E-cadherin expression was an independent prognostic factor for disease-free and overall survival. Conversely, most of the studies published thereafter with larger cohorts failed to demonstrate independent association between E-cadherin expression and disease recurrence after RNU [10–13].

Consistent with the literature [10–13], we found, in our subgroup analysis that, E-cadherin failed to demonstrate any independent prognostic value, outlining its strong association with other established pathological prognostic factors. We found a significant association between E-cadherin expression and adverse clinicopathological features such as advanced pathological tumor stage, high pathological tumor grade, lymph node metastases, LVI, concomitant carcinoma in situ, multifocality, tumor necrosis and sessile architecture. All these factors have been independently associated with worse outcome in UTUC [1, 2, 19, 20, 28–30].

The biological and clinical roles of the E-cadherin-related pathways in urothelial carcinomas are yet to be understood. Indeed, the regulation of E-cadherin is linked to many different biomarkers [2, 3]. Some of them have been already assessed in UTUC. Among them, Snail, a transcription factor is thought to repress the transcription of E-cadherin by binding to elements found in the E-cadherin promoter [12]. An increased Snail expression has been reported as an independent prognostic predictor of recurrence-free and CSS [12]. During EMT, it is thought that transcriptional regulation results in suppression of epithelial markers and gain of mesenchymal markers [7, 12]. This process recognized in different types of cancer including bladder cancer [31, 32] and UTUC [11] has been observed between epithelial E-cadherin and mesenchymal N-cadherin and has been termed the “cadherin switch.” The novel genotype results in an alteration of normal tissue architecture and high-grade, invasive tumors. To our knowledge, only one study by Muramaki et al. [11] addressed the role of N-cadherin expression in UTUC: In this study including 59 patients, N-cadherin expression was an independent prognostic factor of intra- and extra-vesical recurrence after RNU. Combining several biomarkers may help characterize the different pathways involved in tumor aggressiveness and create a prediction algorithm that would improve prognostication, clinical outcome and thus patient survival [33]. At this time the ideal combination of biomarkers remains unfortunately elusive. From the bladder cancer literature and some preliminary upper urinary tract literature, cell cycle markers (p53, pRB, p21, p27 and cyclins), apoptosis markers (Fas, caspase-3, Bcl-2 and survivin) and proliferation markers (Ki67) may be used for a combined approach [34]. Snail expression, N-cadherin expression, AKT pathway, β- or γ-catenins and matrix metalloproteinases are molecular markers associated with EMT that could be analyzed in a combined approach with E-cadherin expression.

We acknowledge that our study has some limitations. First and foremost are those related to its retrospective nature and the immunohistochemical technique. Indeed, this latter technique may be associated with a lack of reproducibility related to the choice of antibodies, the specimen handling procedures, technical demands and scoring protocols. However, tissue microarray with staining protocols and automated scoring systems based on bright field microscopy imaging coupled with advanced color detection software were used to overcome these common limitations. Finally, decreased expression of E-cadherin was defined according to a standard cutoff used in UCB and use of new thresholds may have led to different conclusions.

## Conclusion

Decreased E-cadherin expression is associated with adverse clinicopathological UTUC features and worse outcomes in univariable analyses. E-cadherin expression is, however, not an independent prognostic factor when adjusted for the effects of established prognostic factors, limiting its use in clinical decision-making regarding prognosis after RNU. If E-cadherin’s association with factors of advanced disease is confirmed on UTUC biopsy specimens, it could be used to help in the clinical decision-making regarding kidney-sparing approaches and/or neo-adjuvant chemotherapy.

## Abbreviations

UTUC: Upper tract urothelial carcinoma
RFS: Recurrence-free survival
CSS: Cancer-specific survival
RNU: Radical nephroureterectomy
UCB: Urothelial carcinoma of the bladder
EMT: Epithelial–mesenchymal transition
LVI: Lymphovascular invasion
CI: Confidence interval
HR: Hazard ratio
